# Genito-Urinary and Rectal Surgery

**Published:** 1899-01

**Authors:** Dyer F. Talley

**Affiliations:** Professor Materia Medica and Therapeutics and Lecturer on Genito-Urinary Surgery, Birmingham Medical College


### GENITO-URINARY AND RECTAL SURGERY.

By DYER F. TALLEY, A.M., M.D.

Professor Materia Medica and Therapeutics and Lecturer on Genito-Urinary Surgery, Birmingham Medical College.

### STRICTURE OF THE URETHRA FOLLOWED BY URINARY INFILTRATION.

In December, 1896, W. W., age 36, was referred to me for treatment. He had suffered several years with a stricture about the pendulous urethra, and during the last few weeks before I saw him he had been unable to pass his water, there being simply an overflow from distention of the bladder. When I examined him there was a swollen condition of the perineum, and the scrotal tissues were enlarged to three or four times their normal size, and even a part of the penis was swollen and boggy. My first effort was to enter the bladder through the urethra by passing a small whalebone filiform. I soon found that this could not be accomplished, as the urethra and penis, in certain portions, was so destroyed by the infiltrated urine that the filiform passed through and appeared on the outside. I was then forced to do a perineal section and enter the bladder without a guide. This being accomplished, all the swollen and boggy tissues were very freely incised; the scrotum was literally cut all to pieces and most of the dartos removed as it was necrotic. These incisions were made so freely that one testicle was exposed. The pus and urine that drained from the incisions was extremely foul, and the patient was very septic at the time of operation; his pulse was 160 and he was bathed in a cold sweat. I was afraid he would not survive the operation in that condition. When I saw him next morning he looked a new man, and was feeling much better, as his bladder was well drained by a large tube, and all the infected tissues were drained freely. This patient improved steadily. In about a week after the operation, several inches of the urethra, with all the surrounding cellular tissues, sloughed away "en masse." This extended from the perineal incision to within an inch and a half or two inches of the meatus.

I had never seen the urethra in its entirety slough away, and was curious to know what would become of the canal—whether it could be induced to remain sufficiently patent for the passage of the urine. The canal was kept open by the frequent passage of sounds, and the perineal wound soon healed, leaving a fistula only at the upper portion of the sloughed urethra.

In a few weeks patient was feeling so well he returned to his work at the furnace, after which he neglected to have the sounds passed at regular intervals. He applied to me several weeks ago saying he wanted the fistula (about two inches from the meatus) closed, which will be done after the urethra is sufficiently dilated.

This case teaches that in urinary infiltration the best chances are given by free incisions and the removal of all necrotic tissue. It also teaches that one can get along without the urethra so long as the cicatrinal canal is kept open for the passage of urine and transmission of seminal fluid. This man's virility is not impaired in the least. He says that intercourse is attended with the same pleasure as before the operation.

In all these cases the normal calibre of the urethra should be as nearly restored as possible.

GONORRHŒA TOO LIGHTLY CONSIDERED.

It is quite common among the laity, especially among those who have never had it, to hear the expression that "clap is no worse than a cold." Evidently this is the general belief, as most subjects when they contract a case either treat themselves or apply to the druggist who always has a remedy that will cure in from three to five days. Some of these cases do recover in two or three weeks, in spite of the treatment, while most of them run on until they reach a stage of chronicity from which it is extremely difficult to recover. Probably a majority of these subjects never take an injection properly and do not get the medicine back to the disease; in many cases this is rather fortunate for the patient, as the medicine might damage the urethral mucous membrane much more than the gonococcus could. This would be especially true in cases where patients are directed to get a dime's worth of bluestone and dissolve in a pint of water for an injection. In this way a patient may get a ten-cent cure and probably later on in life it will cost him more than four or five hundred dollars to have strictures treated.

Many physicians consider gonorrhœa easily cured, and some acute cases do respond very nicely to treatment, but the cases that apply to the doctor have usually been self-treated, then treated by the druggist, and by the time they reach the physician, they have become chronic, and there are few, if any, curable diseases so intractable as chronic gonorrhœa. Many cases are treated for months and months until they are lulled into a quiescent condition called a cure, only to be relighted by the first alcoholic or sexual indulgence. This recurrence, fortunately for the doctor's reputation and purse, is called a new case. Many cases of gonorrhœa are thoroughly cured by injections of various astringent and antiseptic solutions with the small syringe, but this is a lazy and negligent way of treating the disease when it can be so much more thoroughly treated by Valentine's irrigation method. When cases apply early for treatment, this plan will usually effect a cure in two weeks, but as most cases are chronic when they reach the doctor, it will generally take as many months.

Those specialists who have treated many cases know how difficult it is to cure chronic gonorrhœa and what dangerous complications may follow a case. I heard one of these specialists in New Orleans remark a few years since that he had rather have syphilis than gonorrhœa.

### THE DISPOSAL OF HEMORRHOIDS.

It is well to know the best methods of getting rid of a condition that causes humanity as much suffering and inconvenience as "piles."

A number of methods have been resorted to and most of them can justly claim a number of cures. Some cases have been relieved by simply opening the pile tumor and letting out the clot—there is usually recurrence after this method and there might be troublesome hemorrhage. Many physicians employ the injection plan of treatment; which consists of injecting some irritating fluid, very often carbolic acid, into the tumor. This method has cured but it is sometimes attended with unpleasant results; it may be the cause of infection and an abscess, or cause the destruction of much more tissue than is necessary to do away with the piles. The ligature method is an excellent one, and in some cases probably the best. It can be relied on as perfectly sure and safe where the ligatures are properly applied. When this method is employed, the mucous membrane around the tumor should be well incised that the ligature may cut through more rapidly and also to avoid the excessive pain that attends cases where this is not done. When the operator has no clamp and cautery, he should always employ the ligature. The clamp and cautery seems almost universally the favorite method now. While in New York recently I was impressed with the success of this operation in very old subjects. At that time Dr. J. P. Tuttle had charge of the almshouse on Blackwell's Island, and there were always some old men from 60 to 80 years of age in the house suffering with piles. Dr. Tuttle operated on about all that came in, and with good results. He has a special forceps to catch up the piles with, which facilitates the operation very much. Two or three catches with this forcep and the clamp and cautery usually completes the operation, which is done very thoroughly and in a very few minutes. There is not so much tissue to slough after this operation, and there are no ligatures in the way to catch fecal matter every time the bowels act, and there is no special pain with the clamp and cautery operation unless the skin is burnt.

The Whitehead operation is very satisfactory where union by first intention is obtained, but extremely unsatisfactory when the stitches break out and the whole circumference of the anus has to granulate. There is always danger of stricture where it has to heal by granulation, and this operation should always be avoided if possible.

